# Neural effects of elevated CO_2_ in fish may be amplified by a vicious cycle

**DOI:** 10.1093/conphys/coz100

**Published:** 2019-12-08

**Authors:** Celia Schunter, Timothy Ravasi, Philip L Munday, Göran E Nilsson

**Affiliations:** 1 Swire Institute of Marine Science and School of Biological Sciences, The University of Hong Kong, Pokfulam, Hong Kong; 2 Marine Climate Change Unit, Okinawa Institute of Science and Technology Graduate University, Tancha, Onna-son, Okinawa 904-0495, Japan; 3 ARC Centre of Excellence for Coral Reef Studies, James Cook University, 1 James Cook Dr, Townsville, Queensland 4814, Australia; 4 Section for Physiology and Cell Biology, Department of Biosciences, University of Oslo, Blindernveien 31, 0316 Oslo, Norway

**Keywords:** Ocean acidification, hypercapnia, GABA, behaviour, fish, brain

## Abstract

Maladaptive behavioural disturbances have been reported in some fishes and aquatic invertebrates exposed to projected future CO_2_ levels. These disturbances have been linked to altered ion gradients and neurotransmitter function in the brain. Still, it seems surprising that the relatively small ionic changes induced by near-future CO_2_ levels can have such profound neural effects. Based on recent transcriptomics data, we propose that a vicious cycle can be triggered that amplifies the initial disturbance, explaining how small pH regulatory adjustments in response to ocean acidification can lead to major behavioural alterations in fish and other water-breathing animals. The proposed cycle is initiated by a reversal of the function of some inhibitory GABA_A_ receptors in the direction of neural excitation and then amplified by adjustments in gene expression aimed at suppressing the excitation but in reality increasing it. In addition, the increased metabolic production of CO_2_ by overexcited neurons will feed into the cycle by elevating intracellular bicarbonate levels that will lead to increased excitatory ion fluxes through GABA_A_ receptors. We also discuss the possibility that an initiation of a vicious cycle could be one of the several factors underlying the differences in neural sensitivity to elevated CO_2_ displayed by fishes.

## Teaser

Lay summaries:

The altered behaviours of fish exposed to elevated CO_2_ have been linked to changes in ion gradients and neurotransmitter function. To explain how relatively small changes in ion concentrations could have such profound neural effects, we propose that a vicious cycle can be triggered that amplifies the initial disturbance.

## Introduction

Since the first reports of behavioural disturbances in fish exposed to elevated CO_2_ (hypercapnia) (Munday *et al*., 2009), a steady stream of reports has shown an array of behavioural and sensory functions being altered by environmental hypercapnia in aquatic animals (Nagelkerken and Munday, 2016; Cattano *et al*., 2018). These impairments, which affect olfactory preferences (by reversing them), lateralization, hearing, vision, learning, physical activity and boldness, occur at elevated CO_2_ levels projected for the next 50 to 100 years and are exhibited not only in fish but also in some invertebrates, including molluscs and crustaceans (Watson *et al.*, 2014; Jellison *et al.,* 2016; Ren *et al.*, 2018). However, there is also a clear intraspecific variation in the response to elevated CO_2_, as we will discuss later.

In order to respond to hypercapnia and defend blood and tissue pH, fish accumulate HCO_3_^−^ and Na^+^ while excreting Cl^−^ and H^+^ (reviewed by Heuer and Grosell, 2014). This occurs both over the gills to defend blood and whole body pH and on the tissue level to protect intracellular pH, and several fish species have been found to preferentially regulate intracellular brain pH when exposed to environmental hypercapnia (Harter *et al.*, 2014; Heuer *et al.*, 2016; Shartau *et al.*, 2016). As a result, the gradients of HCO_3_^−^ and Cl^−^ over cell membranes are likely to be altered, and Nilsson *et al*., (2012) suggested that the neuronal disturbances underlying the behavioural impairments are likely linked to an alteration in the gradients of Cl^−^ and HCO_3_^−^ over neuronal membranes induced by the pH regulatory adjustments. More specifically, it was suggested that these altered gradients can cause a reversal of the function of GABA_A_ receptors in the brain (Nilsson *et al.*, 2012). The GABA_A_ receptor is the major inhibitory neurotransmitter receptor in vertebrates as well as many invertebrates (Tsang *et al.*, 2007). It is an ion channel with permeability for Cl^−^ and HCO_3_^−^, and it is about 2–5 times more permeable to Cl^−^ than to HCO_3_^−^ dependent on brain region and species (Farrant and Kaila, 2007). The electrochemical driving forces for Cl^−^ and HCO_3_^−^ are usually such that Cl^−^ will move inwards while HCO_3_^−^ will move outwards (Staley *et al*., 1995; Farrant and Kaila, 2007), and the opening of this ion channel normally leads to a net influx of negative charge carried by Cl^−^ over the cell membrane, causing hyperpolarization and therefore inhibition of neuronal activity. However, if intracellular levels of HCO_3_^−^ increase and/or extracellular Cl^−^ decrease, the activity of this receptor can turn from inhibitory to excitatory by causing a net outflow of negative charge primarily driven by HCO_3_^−^ (Staley *et al*., 1995; Farrant and Kaila, 2007; Do-Young *et al.*, 2009).

Evidence for an involvement of the GABA_A_ receptor has relied on pharmacological suppression of the receptor with gabazine, a treatment that Nilsson *et al*., (2012) and subsequent studies found to reverse hypercapnia-induced behavioural disturbances in both fish (e.g. Chivers *et al.*, 2014; Chung *et al*., 2014; Lai *et al*., 2015; Lopes *et al.*, 2016; Regan *et al*., 2016) and invertebrates (e.g. Watson *et al.*, 2014). To date, a reversal of the function of GABA_A_ receptors remains the only well-founded mechanistic explanation for the behavioural alterations seen in animals exposed to near-future CO_2_ levels (Tresguerres and Hamilton, 2017). Although measured changes in tissue Cl^−^ and HCO_3_^−^ at 1900 μatm are supportive of the GABA_A_ hypothesis (Heuer *et al.*, 2016), it has surprised many physiologists that the relatively small ionic changes expected in response to projected future pCO_2_ can have such dramatic effects of neural functions (typically seen around 1000 μatm or even lower). It is of course possible that other neural mechanisms are involved in the behavioural alterations seen in fish exposed to elevated CO_2_. For example, Tresguerres and Hamilton (2017) made the hypothetical suggestion that ‘a theoretical OA-induced decrease in glutamate release could be “restored” by gabazine, and this could be erroneously interpreted as GABA_A_ receptor reversal’. However, no plausible mechanistic link between elevated CO_2_ and altered neural function has been proposed for such alternative explanations. Glutamate receptors, for example, are linked to Ca^2+^ and/or Na^+^ channels, so unlike the GABA_A_ receptor that gates Cl^−^ and HCO_3_^−^ fluxes, there is no obvious reason why they would be affected by pH regulatory responses to elevated CO_2_. Similarly, altered electrical responses of the olfactory organ seen during acute high-CO_2_ exposure in sea bass (*Dicentrarchus labrax*) (Porteus *et al.*, 2018) cannot explain altered behaviours in high-CO_2_-exposed fish when the actual behavioural trials are carried out in low-CO_2_ control water (subsequent to the high-CO_2_ exposure) (e.g. Munday *et al.*, 2009, 2016, Dixson *et al*., 2010); and a range of other behaviours and sensory systems not associated with olfaction are also affected by elevated CO_2_ (Heuer and Grosell, 2014; Nagelkerken and Munday, 2016). Moreover, the behavioural disturbances linger on for several days after multiday exposure to elevated CO_2_, as, for example, in studies where CO_2_-treated fish have been put back into their marine habitat (Munday *et al.*, 2010; Chivers *et al.*, 2014). Indeed, the persistence of the effects, together with the fact that it takes several days for the behavioural disturbances to set in (Munday *et al.*, 2010), point at the possibility that processes such as altered gene transcription also could be coming into play.

### Evidence from the transcriptome

**Figure 1:**
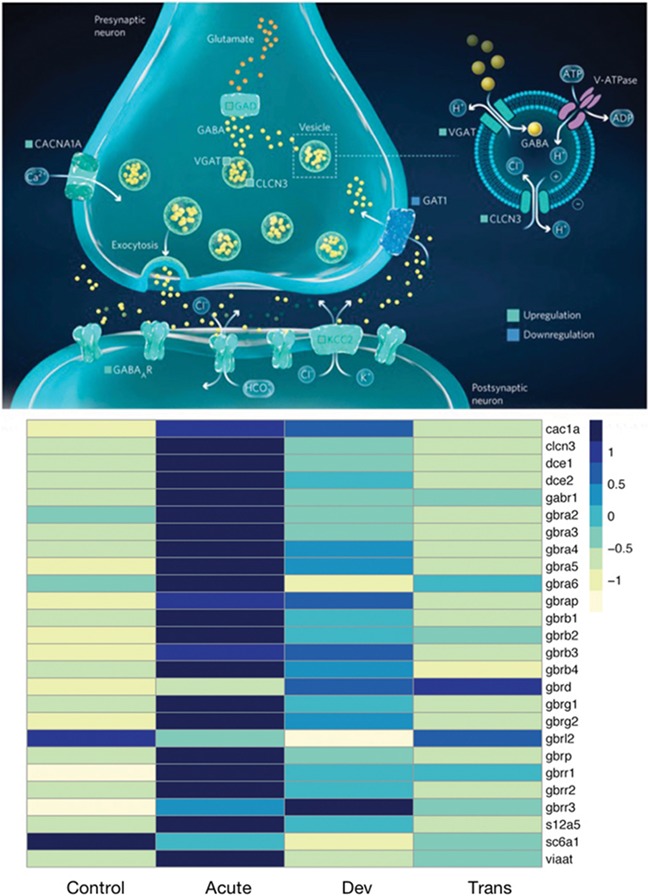
(a) The GABA (γ-aminobutyric acid) signalling pathway in the synapse including differential expression of genes after exposure to elevated CO_2_. GAD = glutamate decarboxylase 1; VGAT = GABA and glycine transporter; CLCN3 = chloride voltage-gated channel 3; KCC2 = neuronal K Cl co-transporter; GAT1 = GABA transporter 1; CACNA1A = brain calcium channel 1; GABAAR = GABA_A_ receptor subunits α,β and γ. From Schunter *et al.* (2018). (b) Heatmap of relative expression levels in *A. polyacanthus* brain after different exposures to elevated CO_2_. Acute: 4-day exposure at the age of 5 months, Developmental (Dev): Exposure since hatching for 5 months, Trans: Transgenerational exposure including parental and 5 months of offspring elevated CO_2_ exposure. Data from Schunter *et al.* (2018) where experimental details are given.

We recently carried out a comprehensive brain transcriptome study on the effect of acute (4 days), developmental (from hatching to 5 months) and transgenerational exposure to hypercapnia (pCO_2_ of 740 μatm) in spiny damselfish (*Acanthochromis polyacanthus*) (Schunter *et al.*, 2018). Importantly, this study provided a strong second line of evidence for an involvement of the GABA_A_ receptor, because a majority of genes linked to the function of this receptor were found to be altered (Fig. 1), particularly after acute and developmental hypercapnia treatment. Based on this transcriptome study, and an analysis of the direction of change of the various components of GABA signalling, we here elaborate on the possibility that altered gene expression leads to a vicious cycle that amplifies the initial disturbance in GABA_A_ receptor function.

Normal function of neural circuits depends on an interplay between excitatory and inhibitory input, and the inhibitory input is generally provided by GABA-releasing neurons affecting GABA_A_ receptors on other neurons (Farrant and Kaila, 2007). Inhibitory and excitatory input has to be balanced by intrinsic regulatory mechanisms. The balancing of excitatory and inhibitory activities has been found to involve adjustments on the transcriptional (mRNA) level, although post-translational changes may also be involved. It has, for example, been repeatedly shown that an overall increase in excitation will lead to transcriptional upregulation of mRNA levels for the different genes making up the subunits of the pentameric GABA_A_ receptor (Uusi-Oukari and Korpi, 2010; Drexel *et al.*, 2013; Yu *et al.*, 2017). Such changes would strive to increase the inhibitory input in overexcited circuits, thereby restoring normal activity levels.

These are exactly the transcriptome signal we find in the brains of spiny damselfish exposed to elevated CO_2_ for 4 days and 5 months (Schunter *et al.*, 2018), suggesting that neural excitation caused by GABA_A_ receptors that have become excitatory triggers regulatory changes in gene expression that strive to restore inhibition by elevating GABA_A_ receptor activity. Indeed, in addition to elevated GABA_A_ receptor expression, we also saw other changes that would serve the same function, including elevated expression of genes responsible for synthesizing GABA (glutamate decarboxylases) and moving it into synaptic vesicles (VGAT and CLCN3). Furthermore, we found reduced expression of mRNA for the transporter protein GAT1 responsible for removing extracellular GABA from synapses (Fig. 1, Supplementary Table 1). Thus, virtually all GABA-associated changes appeared to be aimed at increasing GABAergic signalling.

### A vicious cycle

**Figure 2:**
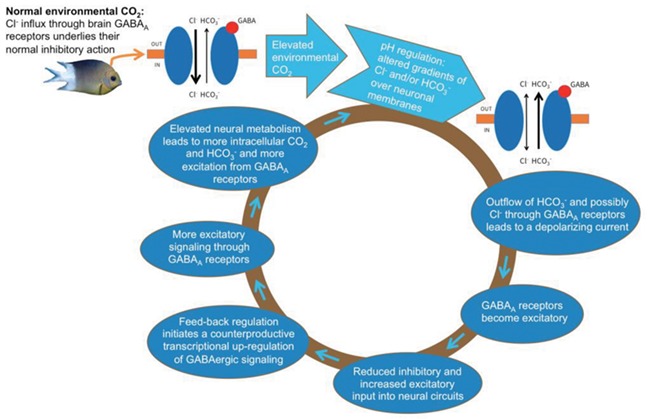
The proposed vicious cycle by which a relatively modest increase in water pCO_2_ can create significant neural and thereby behavioural impairments in fish. pH regulatory mechanisms that compensate for the elevated CO_2_ lead to altered neuronal gradients of Cl^−^ and HCO_3_^−^_._ The cycle is initially triggered by these altered membrane ion gradients that turn GABA_A_ receptors from acting inhibitory (hyperpolarizing) to excitatory (depolarizing). The resultant loss of inhibitory input causes overactivity in neuronal circuits that leads to transcriptomal changes striving to boost inhibitory GABA signalling. Unfortunately, this becomes counterproductive since GABA_A_ receptors have become excitatory, and the result is even more excitatory overactivity. The increase in electric activity leads to higher energy demands and therefore increased metabolic production of intracellular CO_2_, which is turned into HCO_3_^−^ by intracellular carbonic anhydrase, thereby raising intracellular [HCO_3_^−^] that will further increase a depolarizing current through GABA_A_ receptors.

Unfortunately, these transcriptional changes are likely to make things worse if GABA_A_ receptors have become excitatory due to altered ion gradients. This is where we suggest that a vicious cycle is triggered, where regulatory processes aimed at increasing inhibition instead cause more excitation (Fig. 2). Importantly, the cycle could be further amplified by the resultant increase in neural metabolic activity. Electrical activity is the main energy consumer in the brain (Lutz *et al.*, 2003), and any increase in excitatory signalling in neural circuits will translate into increased energy metabolism and thereby metabolic production of CO_2_ inside neurons. Indeed, this strong link between electrical activity and metabolism in brain tissue is the reason why techniques such as PET scanning and functional MRI can be used to detect changes in brain activity. Because neurons contain high levels of carbonic anhydrase (Ruusuvuori and Kaila, 2014), which catalyses the conversion of CO_2_ and H_2_O into HCO_3_^−^, it is likely that intracellular HCO_3_^−^ levels will rise when neurons become overactive and produce more metabolically derived CO_2_. An elevation of intracellular HCO_3_^−^, which will flow out of GABA_A_ receptors and counteract or overwhelm the influx of Cl^−^ (Staley *et al*., 1995; Farrant and Kaila, 2007; Do-Young *et al.*, 2009), would further drive GABA_A_ receptors in the direction of excitation. Indeed, this could in itself drive a vicious cycle also in the absence of changes in GABA-related gene expression. Interestingly, both Schunter *et al.* (2018) and Williams *et al.* (2018) found increased expression of glucose transporters in the brain of fish exposed to elevated CO_2_, which is indicative of increased neural metabolism.

There are two other studies on transcriptional changes in fish brains in response to elevated CO_2_. Both show changes in the direction of increased GABAergic signalling although not as many of the components appear altered as in the spiny damselfish. Thus, a study on stickleback (*Gasterosteus aculeatus*) behaviourally affected by elevated CO_2_ found elevated mRNA levels of GABA_A_ alpha-subunits, which are major subunits present in all GABA_A_ receptors (Lai *et al*., 2016). Another recent study showed changes in GABA-related gene expression (mRNA) in the olfactory bulb of ocean-phase coho salmon (*Oncorhynchus kisutch*) exposed to elevated CO_2_ (Williams *et al*., 2018). In the latter study, stimulated GABAergic signalling was indicated by reduced mRNA for a transporter responsible for GABA reuptake (as also seen in the spiny damselfish), while GABA_A_ receptor subunits appeared unaffected. Reduced GABA reuptake would lead to elevated extracellular GABA levels that will activate GABA_A_ receptors. However, Williams *et al*. (2018) found that GABA_B_ receptor expression was upregulated. An upregulation of GABA_B_ receptors could help dampen a vicious cycle in this species as GABA_B_ receptors are not anion channels but act in an inhibitory way by opening K^+^ channels that are less likely to be affected by pH regulatory responses to elevated CO_2_.

Obviously, more studies will be needed to show if these transcriptional responses are as widespread among fish, and possibly invertebrates, as the behavioural alterations caused by environmental hypercapnia. It may be that a vicious cycle fuelled by altered gene expression and increased metabolic CO_2_ production is more developed in some species or life stages than others and that this correlates with their neural sensitivity to elevated CO_2_ exposure.

Notably, the pattern of altered gene expression that we found was absent or largely subdued in spiny damselfish where both the parents and offspring were exposed to elevated CO_2_ levels (Schunter *et al.*, 2016, 2018). Because behavioural disturbances have been found to persist even after such transgenerational CO_2_ exposure (Welch *et al.*, 2014), it is tempting to speculate that the altered GABAergic function has at this stage entered a chronic phase that is no longer reflected at the level of mRNA expression. At this stage, a vicious cycle could still be driven by elevated intracellular levels of HCO_3_^−^ derived from increased neural excitation and metabolism.

### Variation among species

The large number of studies that have now been conducted into behavioural effects of elevated CO_2_ in fish show that there is considerable interspecific variation in sensitivity (Ferrari *et al.*, 2011; Schmidt *et al.,* 2017a) and some species do not seem to be affected at all (Maneja *et al.*, 2013; Jutfelt and Hedgärde, 2015; Heinrich *et al.*, 2016, Kwan *et al.,* 2017).

One would expect that the alteration of ion gradients needs to reach a threshold for triggering a self-amplifying vicious cycle altering GABAergic function. Nilsson *et al.* (2012) suggested that species and life stages with high metabolic rates, and therefore large respiratory surface areas and high rates of gas exchange, could be particularly prone to have their tissue ion gradients altered by elevated CO_2_ levels. The rapid CO_2_ flux over a large respiratory surface area could bring the internal CO_2_ levels closer to those of the water, making the animal more affected by any changes in water pCO_2_. Indeed, tropical coral reef fish larvae, where the behavioural effects of elevated CO_2_ were first observed, display extremely high rates of gas exchange (Nilsson *et al.*, 2007). One could argue that the high rate of metabolic CO_2_ production in highly active fishes also would lead to high internal levels of CO_2_ and HCO_3_^−^. However, because of the high solubility of CO_2_ in water, it is more easily released over the gills than O_2_ is taken up. Indeed, one of the lowest blood CO_2_ levels recorded in fish has been found in mackerel (*Scomber scombrus*), especially when swimming at high speed (i.e. at high metabolic CO_2_ production rates) where blood pCO_2_ approached 1 mm Hg (Boutilier *et al.*, 1984). However, other factors are likely to be involved as closely related species may show clear differences in sensitivity to elevated CO_2_ (Ferrari *et al.*, 2011; Schmidt *et al.*, 2017a). One factor that could underlie species and life stage differences is adaptation to naturally occurring variation in pCO_2_ in the habitats, allowing for resilience to any CO_2_-induced alterations in ion gradients and GABA_A_ receptor function. Indeed, many of the species exhibiting behavioural tolerance to elevated CO_2_ occupy habitats that periodically experience naturally high pCO_2_ (Heinrich *et al.*, 2016, Kwan *et al.*, 2017). For example, studies have indicated that Atlantic cod (*Gadus morhua*) are not behaviourally affected by elevated CO_2_ (Jutfelt and Hedgärde, 2015; Maneja *et al.*, 2013; Schmidt *et al.,* 2017a). By contrast, behavioural lateralization was affected by elevated CO_2_ in polar cod (*Boreogadus saida*) (Schmidt *et al.,* 2017a). A possible explanation for this difference is that Atlantic cod are adapted to a large natural variation in pCO_2_ in the habitats they commonly occupy and thus have different GABAergic responses to high CO_2_ (Schmidt *et al.,* 2017b). Another possible factor here could involve differences in the acid–base regulatory capacity between the species, where those that show a low regulatory response to acidification, and therefore essentially maintain internal HCO_3_^−^ concentrations and gradients, will retain normal GABAergic function. However, failure to defend internal pH could bring other problems. For example, it is noteworthy that Atlantic cod is one of the few species of fish for which projected future CO_2_ levels have been found to directly impact larval development (Frommel *et al.,* 2012) and greatly increase mortality (Stiasny *et al.,* 2016). A weak acid–base regulatory response in larval Atlantic cod could potentially explain both their high mortality in high CO_2_ compared with other fishes and the absence of behavioural effects, because the changes in Cl^−^ and HCO_3_^−^ could be too small to induce a significant change in GABAergic function.

## Conclusions and directions for the future

To conclude, the vicious cycle that we describe here can explain why relatively small initial disturbances in neuronal ion gradients after a few days become manifested in neural dysfunction severe enough to cause an array of behavioural alterations. A self-amplifying cycle that involves changes in gene expression and ultimately protein synthesis may take some time to be fully expressed, which can explain why the behavioural disturbances are not seen until after 2–3 days of high-CO_2_ exposure and lingers on for several days after the exposure has ended (Munday *et al.*, 2010). Moreover, if a sustained increase in water pCO_2_ is needed to trigger and/or sustain the vicious cycle, then this could also explain recent findings showing that daily cycling of water pCO_2_ has less behavioural effects in fish than a maintained high CO_2_ level (Jarrold *et al.*, 2017).

The variation between species and life stages in CO_2_ responses should prove useful for testing hypotheses about the relationship between altered behaviours and changes in GABAergic function caused by elevated CO_2_. More comparative studies on more species and life stages would of course be most welcome as they would allow us to better identify and examine the patterns and processes that determine the neural sensitivity to elevated CO_2_. Experimentally, finding signs of the vicious circle we propose here could involve transcriptomic and proteomic studies. It would be desirable if these could be linked to measurements of ion and pH regulatory variables in blood and brain tissue, although this will be difficult in small individuals. Future studies could adopt new technologies that allow a more fine-scale approach, such as single-cell transcriptomics to examine the responses of specific cell types in discrete brain regions rather than whole brain tissue. Direct electrophysiological recordings of effects of elevated CO_2_ on neural functions, which have only rarely been done (Chung *et al.*, 2014; Porteus *et al.*, 2018; Williams *et al.*, 2018), will also bring us closer to understanding the mechanisms involved.

Understanding the physiological mechanisms by which elevated CO_2_ affects fish behaviour, and how this links to variation in responses among species and habitats, will help establish where and when future ocean acidification conditions could impact fish populations. For iconic and/or high value species, such as salmon (Ou *et al.*, 2015; Williams *et al.*, 2018), this knowledge might even be used in adaptive management practices to limit exposure to the precise conditions that induce behavioural effects. A detailed physiological understanding might also be exploited in aquaculture to manipulate CO_2_ levels such that any effects on behaviour are optimal for production.

## Supplementary Material

Schunter_et_al_Suppl_Table1_coz100

## References

[ref1] Boutilier RG, Aughton P, Shelton G (1984) O_2_ and CO_2_ transport in relation to ventilation in Atlantic mackerel, Scomber scombrus. *Can J Zool* 62: 546–554.

[ref2] Cattano C, Claudet J, Domenici P, Milazzo M (2018) Living in a high CO_2_ world: a global meta-analysis shows multiple trait-mediated fish responses to ocean acidification. *Ecole Monogr* 88:ecm 1297.

[ref3] Chivers DP, McCormick MI, Nilsson GE, Munday PL, Watson SA, Meekan MG, Mitchell MD, Corkill KC, Ferrari MCO (2014) Impaired learning of predators and lower prey survival under elevated CO_2_: a consequence of neurotransmitter interference. *Glob Chang Biol* 20: 515–522. 23765546 10.1111/gcb.12291

[ref4] Chung W-S, Marshall NJ, Watson SA, Munday PL, Nilsson GE (2014) Ocean acidification slows retinal function in a damselfish through interference with GABA_A_ receptors. *J Exp Biol* 217: 323–326. 24477607 10.1242/jeb.092478

[ref5] Dixson DL, Munday PL, Jones GP (2010) Ocean acidification disrupts the innate ability of fish to detect predator olfactory cues. *Ecol Lett* 13: 68–75. 19917053 10.1111/j.1461-0248.2009.01400.x

[ref6] Do-Young K, Fenoglio KA, Kerrigan JF, Rho JM (2009) Bicarbonate contributes to GABA_A_ receptor-mediated neuronal excitation in surgically-resected human hypothalamic hamartomas. *Epilepsy Res* 83: 89–93. 19022626 10.1016/j.eplepsyres.2008.09.008 PMC2692870

[ref7] Drexel M, Kirchmairand E, Sperk G (2013) Changes in the expression of GABA_A_ receptor subunit mRNAs in parahippocampal areas after kainic acid-induced seizures. *Front Neural Circuits* 7: article 142. 10.3389/fncir.2013.00142 PMC3776158 24065890

[ref8] Farrant M, Kaila K (2007) The cellular, molecular and ionic basis of GABA_A_ receptor signaling. *Prog Brain Res* 160: 59–87. 17499109 10.1016/S0079-6123(06)60005-8

[ref9] Ferrari MCO, Dixson DL, Munday PL, McCormick MI, Meekan MG, Sih A, Chivers DP (2011) Intrageneric variation in antipredator responses of coral reef fishes affected by ocean acidification: implications for climate change projections on marine communities. *Glob Chang Biol* 17: 2980–2986.

[ref10] Frommel AY, Maneja R, Lowe D, Malzahn AM, Geffen AJ, Folkvord A, Piatkowski U, Reusch TBH, Clemmesen C (2012) Severe tissue damage in Atlantic cod larvae under increasing ocean acidification. *Nat Clim Chang* 2: 42–46.

[ref11] Harter ST, Shartau RB, Baker DW, Jackson DC, Val AL, Brauner CJ (2014) Preferential intracellular pH regulation represents a general pattern of pH homeostasis during acid–base disturbances in the armoured catfish, *Pterygoplichthys pardalis*. *J Comp Physiol B* 184: 709–718. 24973965 10.1007/s00360-014-0838-8

[ref12] Heinrich DDU, Watson SA, Rummer JL, Brandl SJ, Simpfendorfer CA, Heupel MR, Munday PL (2016) Foraging behaviour of the epaulette shark *Hemiscyllium ocellatum* is not affected by elevated CO_2_. *ICES J Mar Sci* 73: 633–640.

[ref13] Heuer RM, Grosell M (2014) Physiological impacts of elevated carbon dioxide and ocean acidification on fish. *Am J Physiol Regul Integr Comp Physiol* 307: R1061–R1084. 25163920 10.1152/ajpregu.00064.2014

[ref14] Heuer RM, Welch MJ, Rummer JL, Munday PL, Grosell M (2016) Altered brain ion gradients following compensation for elevated CO2 are linked to behavioural alterations in a coral reef fish. *Sci Rep* 6: 33216. 10.1038/srep33216 PMC5020430 27620837

[ref15] Jarrold MD, Humphrey C, McCormick MI, Munday PL (2017) Diel CO_2_ cycles reduce severity of behavioural abnormalities in coral reef fish under ocean acidification. *Sci Rep* 7: 10153. 10.1038/s41598-017-10378-y PMC5578974 28860652

[ref16] Jellison BM, Ninokawa AT, Hill TM, Sanford E, Gaylord B (2016) Ocean acidification alters the response of intertidal snails to a key sea star predator. *Proc R Soc B-Biol Sci* 283: 20160890. 10.1098/rspb.2016.0890 PMC4936044 27358371

[ref17] Jutfelt F, Hedgärde M (2015) Juvenile Atlantic cod behavior appears robust to near-future CO_2_ levels. *Front Zool* 12: 11. 27408612 10.1186/s12983-015-0104-2 PMC4940919

[ref18] Kwan GT, Hamilton TJ, Tresguerres M (2017) CO_2_-induced ocean acidification does not affect individual or group behaviour in a temperate damselfish. *R Soc Open Sci* 4: 170283. 28791154 10.1098/rsos.170283 PMC5541549

[ref19] Lai F, Jutfelt F, Nilsson GE (2015) Altered neurotransmitter function in CO_2_-exposed stickleback (*Gasterosteus aculeatus*): a temperate model species for ocean acidification research. *Conserv Physiol* 3: cov018. 10.1093/conphys/cov018 PMC4778464 27293703

[ref20] Lai F, Fagernes CE, Jutfelt F, Nilsson GE (2016) Expression of genes involved in brain GABAergic neurotransmission in three-spined stickleback exposed to near-future CO_2_. *Conserv Physiol* 4: cow068. 10.1093/conphys/cow068 PMC5196030 28066553

[ref21] Lopes AF, Morais P, Pimentel M, Rosa R, Munday PL, Gonçalves EJ, Faria AM (2016) Behavioural lateralization and shoaling cohesion of fish larvae altered under ocean acidification. *Mar Biol* 163: 243.

[ref22] Lutz PL, Nilsson GE, Prentice HM (2003) The Brain Without Oxygen. Kluwer Academic Publishers, Dordrecht.

[ref23] Maneja RH, Frommel AY, Browman HI, Clemmesen C, Geffen AJ, Folkvord A, Piatkowski U, Durif CMF, Bjelland R, Skiftesvik AB (2013) The swimming kinematics of larval Atlantic cod, *Gadus morhua* L., are resilient to elevated seawater pCO_2_. *Mar Biol* 160: 1963–1972.

[ref24] Munday PL, Dixson DL, Donelson JM, Jones GP, Pratchett MS, Devitsina GV, Døving KB (2009) Ocean acidification impairs olfactory discrimination and homing ability of a marine fish. *Proc Natl Acad Sci U S A* 106: 1848–1852. 19188596 10.1073/pnas.0809996106 PMC2644126

[ref25] Munday PL, Dixson DL, McCormick MI, Meekan M, Ferrari MCO, Chivers DP (2010) Replenishment of fish populations is threatened by ocean acidification. *Proc Natl Acad Sci U S A* 107: 12930–12934. 20615968 10.1073/pnas.1004519107 PMC2919925

[ref26] Munday PL, Welch MJ, Allan BJM, Watson S-A, McMahon SJ, McCormick MI (2016) Effects of elevated CO_2_ on predator avoidance behaviour by reef fishes is not altered by experimental test water. *PeerJ* 4: e2501. 10.7717/peerj.2501 PMC5068342 27761317

[ref27] Nagelkerken I, Munday PL (2016) Animal behaviour shapes the ecological effects of ocean acidification and warming: moving from individual to community-level responses. *Glob Chang Biol* 22: 974–989. 26700211 10.1111/gcb.13167

[ref28] Nilsson GE, Östlund-Nilsson S, Penfold R, Grutter AS (2007) From record performance to hypoxia tolerance: respiratory transition in damselfish larvae settling on a coral reef. *Proc R Soc B-Biol Sci* 274: 79–85. 10.1098/rspb.2006.3706 PMC1679883 17015334

[ref29] Nilsson GE, Dixson DL, Domenici P, McCormick MI, Sorensen C, Watson SA, Munday PL (2012) Near-future carbon dioxide levels alter fish behaviour by interfering with neurotransmitter function. *Nat Clim Chang* 2: 201–204.

[ref30] Ou M, Hamilton TJ, Eom J, Lyall EM, Gallup J, Jiang A, Lee J, Close DA, Yun S-S, Brauner CJ (2015) Responses of pink salmon to CO_2_-induced aquatic acidification. *Nat Clim Chang* 5: 950–955.

[ref31] Porteus CS, Hubbard PC, Webster TMU, van Aerl R, Canário AVM, Santos EM, Wilson RW (2018) Near-future CO2 levels impair the olfactory system of a marine fish. *Nat Clim Chang* 8: 737–743.

[ref32] Regan MD et al. (2016) Ambient CO_2_, fish behaviour and altered GABAergic neurotransmission: exploring the mechanism of CO_2_-altered behaviour by taking a hypercapnia dweller down to low CO_2_ levels. *J Exp Biol* 219: 109–118. 26739687 10.1242/jeb.131375

[ref33] Ren Z, Mu C, Li R, Song W, Wang C (2018) Characterization of a gamma-aminobutyrate type a receptor-associated protein gene, which is involved in the response of *Portunus trituberculatus* to CO2-induced ocean acidification. *Aquac Res* 49: 2393–2403.

[ref34] Ruusuvuori E, Kaila K (2014) Carbonic anhydrases and brain pH in the control of neuronal excitability. *Subcell Biochem* 75: 271–290. 24146384 10.1007/978-94-007-7359-2_14

[ref35] Schmidt M, Gerlach G, Leo E, Kunz K, Swoboda S, Pörtner H-O, Bock C, Storch D (2017a) Impact of ocean warming and acidification on the behaviour of two co-occurring gadid species, *Boreogadus saida* and *Gadus morhua*, from Svalbard. *Mar Ecol Prog Ser* 571: 183–191.

[ref36] Schmidt M, Windisch HS, Ludwichowski K-U, Seegert SLL, Pörtner H-O, Storch D, Bock C (2017b) Differences in neurochemical profiles of two gadid species under ocean warming and acidification. *Front Zool* 14: 49. 29093740 10.1186/s12983-017-0238-5 PMC5661927

[ref37] Schunter C, Welch MJ, Ryu T, Zhang H, Berumen ML, Nilsson GE, Munday PL, Ravasi T (2016) Molecular signatures of transgenerational response to ocean acidification in a species of reef fish. *Nat Clim Chang* 6: 1014–1018.

[ref38] Schunter C, Welch MJ, Nilsson GE, Rummer JL, Munday PL, Ravasi T (2018) An interplay between plasticity and parental phenotype determines impacts of ocean acidification on a reef fish. *Nat Ecol Evol* 2: 334–342. 29255298 10.1038/s41559-017-0428-8

[ref39] Shartau RB, Baker DW, Crossley IIDA, Brauner CJ (2016) Preferential intracellular pH regulation: hypotheses and perspectives. *J Exp Biol* 219: 2235–2244. 27489212 10.1242/jeb.126631

[ref40] Staley KJ, Soldo BL, Proctor WR (1995) Ionic mechanisms of neuronal excitation by inhibitory GABA_A_ receptors. *Science* 269: 977–981. 7638623 10.1126/science.7638623

[ref41] Stiasny MH, Mittermayer FH, Sswat M, Voss R, Jutfelt F, Chierici M, Puvanendran V, Mortensen A, Reusch TBH, Clemmesen C (2016) Ocean acidification effects on Atlantic cod larval survival and recruitment to the fished population. *PLoS One* 11: e0155448. 10.1371/journal.pone.0155448 PMC4995109 27551924

[ref42] Tresguerres M, Hamilton TJ (2017) Acid–base physiology, neurobiology and behaviour in relation to CO_2_-induced ocean acidification. *J Exp Biol* 220: 2136–2148. 28615486 10.1242/jeb.144113

[ref43] Tsang S-Y, Ng S-K, Xu Z, Xue H (2007) The evolution of GABA_A_ receptor–like genes. *Mol Biol Evol* 24: 599–610. 17135332 10.1093/molbev/msl188

[ref44] Uusi-Oukari M, Korpi ER (2010) Regulation of GABA_A_ receptor subunit expression by pharmacological agents. *Pharmacol Rev* 62: 97–135. 20123953 10.1124/pr.109.002063

[ref45] Watson S-A, Lefevre S, McCormick MI, Domenici P, Nilsson GE, Munday PL (2014) Marine mollusc predator-escape behaviour altered by near-future carbon dioxide levels. *Proc R Soc B-Biol Sci* 281: 20132377. 10.1098/rspb.2013.2377 PMC3843832 24225456

[ref46] Welch MJ, Watson S-A, Welsh JQ, McCormick MI, Munday PL (2014) Effects of elevated CO_2_ on fish behaviour undiminished by transgenerational acclimation. *Nature*. *Clim Chang* 4: 1086–1089.

[ref47] Williams CR, Dittman AH, McElhany P, Busch DS, Maher MT, Bammler TK, MacDonald JW, Gallagher EP (2018) Elevated CO_2_ impairs olfactory-mediated neural and behavioral responses and gene expression in ocean-phase coho salmon (*Oncorhynchus kisutch*). *Glob Chang Biol*: gcb.14532. 10.1111/gcb.14532 PMC7065673 30561876

[ref48] Yu J, Liu L, Wang L, Wu G, Wu M (2017) Increased GABA (a) receptors α1, γ2, δ subunits might be associated with the activation of the CREB gene in low Mg^2+^ model of epilepsy. *Neuropsychiatry* 7: 398–405.

